# *Quo Vadis* Nordic Hamstring Exercise-Related Research?—A Scoping Review Revealing the Need for Improved Methodology and Reporting

**DOI:** 10.3390/ijerph191811225

**Published:** 2022-09-07

**Authors:** Tobias Alt, Jannik Severin, Marcus Schmidt

**Affiliations:** 1 Department of Biomechanics, Performance Analysis and Strength & Conditioning, Olympic Training and Testing Centre Westphalia, 44139 Dortmund, Germany; 2 Institute of Movement and Neuroscience, German Sport University, 50933 Cologne, Germany; 3 Institute for Sport and Sport Science, TU Dortmund University, 44227 Dortmund, Germany

**Keywords:** eccentric resistance training, knee flexor strength, hamstring injury prevention, neuromuscular adaptations, execution quality, Nordic Curls, hamstring lowers

## Abstract

The objective of this scoping review is to assess Nordic Hamstring Exercise quality (ANHEQ) of assessments and interventions according to the ANHEQ rating scales and to present practical recommendations for the expedient design and reporting of future studies. A total of 71 Nordic Hamstring Exercise (NHE) assessments and 83 NHE interventions were selected from the data sources PubMed, Scopus, and SPORTDiscus. Research studies which were presented in peer-reviewed academic journals and implemented the NHE during laboratory-based assessments or multi-week interventions met the eligibility criteria. NHE assessments analyzed force (51%), muscle activation (41%), knee angle kinematics (38%), and bilateral symmetry (37%). NHE interventions lasted 4–8 weeks (56%) and implied an exercise volume of two sessions per week (66%) with two sets per session (41%) and ≥8 repetitions per set (39%). The total ANHEQ scores of the included NHE assessments and interventions were 5.0 ± 2.0 and 2.0 ± 2.0 (median ± interquartile range), respectively. The largest deficits became apparent for consequences of impaired technique (87% 0-point-scores for assessments) and kneeling height (94% 0-point-scores for interventions). The 0-point-scores were generally higher for interventions compared to assessments for rigid fixation (87% vs. 34%), knee position (83% vs. 48%), kneeling height (94% vs. 63%), and separate familiarization (75% vs. 61%). The single ANHEQ criteria, which received the highest score most frequently, were rigid fixation (66% of assessments) and compliance (33% of interventions). The quality of NHE assessments and interventions was generally ‘below average’ or rather ‘poor’. Both NHE assessments and interventions suffered from imprecise reporting or lacking information regarding NHE execution modalities and subsequent analyses. Based on the findings, this scoping review aggregates practical guidelines how to improve the design and reporting of future NHE-related research.

## 1. Introduction

First introduced in 1880 [1], the Nordic Hamstring Exercise (NHE)—also referred to as Russian Hamstrings/Curls or Nordic (Hamstring) Lowers/Curls—has evolved into a popular resistance training exercise to selectively improve knee flexor strength and to efficiently prevent knee and hamstring injuries. Two recent systematic reviews and meta-analyses independently proved that the regular implementation of this supramaximal, body-weight, open-kinetic chain exercise caused an average reduction of hamstring strain injuries of 51% [2,3]. Additionally, evidence-based best-practice guidelines recommend the implementation of the NHE as an inherent part of preventive training regimen for anterior cruciate ligament injuries [4].

Despite strong empirical evidence favoring the NHE for eccentric knee flexor strengthening [5,6,7,8,9], injury risk mitigation [2,3,10,11,12,13,14,15,16,17], fascicle lengthening [5,7,8,18,19,20,21,22,23], and performance enhancement of sprints [24,25,26,27,28,29,30] and jumps [28,29,31,32,33,34], the exercise does not receive complete approval and appreciation by practitioners and scientists. This is particularly attributable to its challenging execution, its bilateral nature, its knee dominance, and its distinctive muscle activation patterns [34,35,36,37,38,39]. The supramaximal nature of the NHE can only be achieved if there is a break point (increased angular velocity) towards the end of the range of motion (ROM).

However, most athletes demonstrate insufficient strength capacities to maintain a high muscle activation at extended knee angles during NHE execution (~30° to 0° knee flexion) [6,39,40,41,42,43,44,45]. Within the second half of the exercise, the gravity-induced resistant moment imposed by the trunk position progressively increases. This elicits very high knee flexor activation at longer muscle lengths, precisely at the most vulnerable injury position [3,40,42,46,47,48]. A premature ending of the controlled eccentric action is a major limitation of current NHE studies to address [11], because consistently high muscle activation in more extended knee angles represents an important target for prevention and rehabilitation [35,48,49,50]. Improving this deficient ability will potentially contribute to reduce the consistently high injury incidences [2,3,35,50,51,52] by enlarging the ‘muscle’s safe operating range’ [53].

In 2004, Mjølsnes et al. [12] introduced a 10-week progressive NHE training regimen, which has become the most replicated intervention program in published research [5,11,13,25,28,29,31,34,36,37,43,54,55,56,57,58,59,60]. However, this particular study suffers from a substantial lack of information about NHE execution quality [61]. Common NHE-related research usually revealed imprecise or deficient information about execution modalities, testing procedures, and data processing as well. Additionally, a current meta-analysis reveals that the evidence underpinning the protective effect of the NHE remains inconclusive due to the high risk of bias of published intervention studies [62]. Therefore, reliable rating scales for assessing Nordic Hamstring Exercise quality (ANHEQ) have been recently introduced to evaluate the informative and scientific value of existing NHE-related evidence and to improve the design and reporting of future NHE studies [61].

Common NHE interventions involved a progression of exercise volume, and the exercise was usually performed on the floor while a partner applied pressure to the heels [10,11,12,13,63]. Since 2018, the focus of NHE interventions has been directed towards an accentuation of exercise intensity [7,8,19,64]. However, high-intensity NHE training can be hardly carried out with the support of a single partner because the partner’s capacity to stabilize may affect the athlete’s ability to maximally perform the exercise and may limit the benefits of the NHE [12,61,65]. Therefore, several devices have been used to ensure a safe NHE execution via a rigid resistance at the heels: the NordBord (from prototype [66] to commercial product [67]), related products [26,48,68,69], several custom-made devices [18,22,44,70,71,72,73,74,75,76,77], usual training equipment [78,79,80], and a dynamometer [6,30].

These devices facilitate standardized procedures of NHE assessments and interventions. Nevertheless, mostly insufficient knee positions and kneeling heights impede a controlled NHE execution across the entire ROM until full knee extension [61]. Usually, information is lacking to which extent participants were able to delay the excessive downward acceleration of their body (the body position at which the individual can no longer resist the sinusoidal increasing gravitational moment so that the movement speed irreversibly increases). However, withstanding high eccentric loads in extended knee angles is crucial to prevent muscle and knee injuries [2,3,4,35,48,49,50]. Therefore, not only the amount of strength is of particular interest, but the controlled ROM during NHE execution (‘angle at downward acceleration’, φ_DWA_) and the maintenance of a high muscle activation [40,41,43,71,81,82,83,84] as well. The combination of both parameters provides a comprehensive evaluation of NHE performance. To our knowledge, appropriate studies are rare, which presented these parameters in conjunction [6].

Available research about the NHE lacks precise information about study design and reporting [61,62,85]. Nevertheless, to the best of our knowledge there is no review article available that has aggregated this information. The aim of the present scoping review was to assess the quality of NHE assessments and interventions according to the ANHEQ rating scales and to answer the following research questions:

1. Which descriptive characteristics (e.g., participants, diagnostic tools, and parameters of NHE assessments and stimulus characteristics of NHE interventions) are used by the included full-text articles?
2. How high are the total ANHEQ scores of previously published NHE assessment and intervention studies?
3. Which ANHEQ items revealed the highest deficits and received the highest score most frequently?
4. How should future ANHEQ-related research guarantee the best possible study design and reporting?

Referring to a previously published small-sized literature analysis [61], we hypothesized that the quality of NHE assessments and interventions is ‘average’ and ‘below average’, respectively. Based on the current literature, we present specific practical guidelines for future NHE studies, which will help practitioners and scientists to improve the quality of both design and reporting.

## 2. Methods

### 2.1. Search Strategy and Study Selection

**Figure 1:**
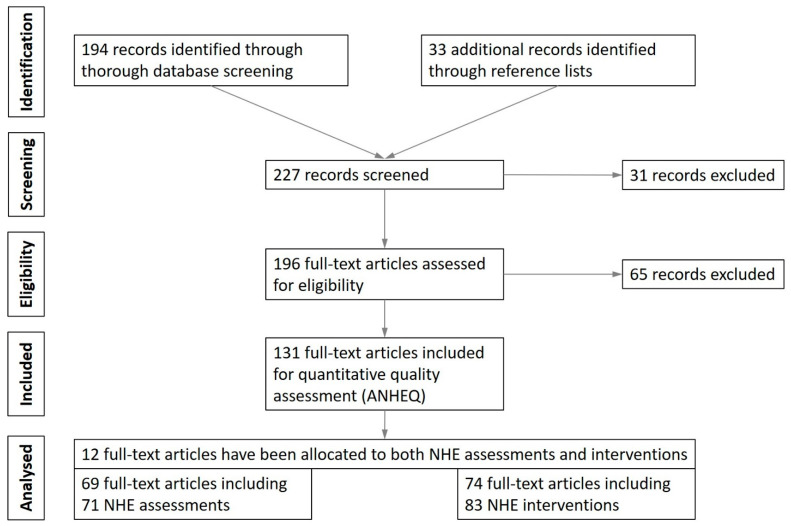
Flow chart of study identification, screening, and selection for the quantitative analysis of the quality of Nordic Hamstring Exercise (NHE) assessments and interventions.

The design of this scoping review was developed according to the guidelines of the PRISMA statement [86]. A review protocol was not pre-registered. To identify relevant articles, a Boolean/phrase search mode was utilized using the keywords Nordic Hamstring Exercise OR Nordic Curls OR Nordic Hamstring Lowers. These keywords were applied to the databases PubMed, Scopus, and SPORTDiscus and were filtered to include studies that were (1) presented in peer-reviewed academic journals, that were (2) written in English, and that (3) examined human subjects. No restrictions were placed upon the age or sex of the subjects. Database entries were searched from the earliest reported date until end of December 2020 (e-pub date). In addition to the electronic database search, the reference lists of the included papers were also cross-checked. Publication title, author(s), and year from all studies identified in the literature search were compiled in an excel spreadsheet, used for further evaluation. Once all results were collected, titles and abstracts were screened for eligibility to identify relevant articles for full-text review and inclusion. A flow chart of study selection is presented in Figure 1.

### 2.2. Eligibility Criteria

The focus of this scoping review was the identification of research studies that implemented dynamic executions of the NHE during laboratory-based assessments or long-term training interventions in any sporting activity. No restrictions were made according to the primary goal of the investigations (e.g., injury prevention). Previously published reviews or meta-analyses were not included. Assessments were eligible if an analysis of NHE performance and/or execution was performed. Studies which implemented the NHE as obligatory or optional part of a multi-exercise training regimen were also included. Studies using the NHE during training interventions had to focus on long-term adaptations, usually involving multi-week protocols, or prospective cohort studies. Investigations which implemented the NHE as acute bout of exercise (e.g., [45]) to induce acute changes of neuromuscular or psycho-physiological status of the human body (e.g., inducing fatigue) without presenting any NHE-related data were not considered for further analysis. If assessments and interventions used or compared several different NHE execution modalities or intervention protocols, separate analyses were conducted. Consequently, the number of analyzed NHE assessments and interventions exceeded the number of included full-text articles (Figure 1). Two investigators (JS and MSu) conducted the initial search, removed duplicates, and checked the remaining papers for eligibility. A third and fourth investigator (TA and MSc) facilitated consensus in case of disagreements about study selection or eligibility. A total of 227 full-text articles was initially identified and screened utilizing the eligibility criteria.

### 2.3. Quality Assessment and Data Analysis

The three authors of this scoping review (TA, JS, and MSc) independently rated each study according to the recently introduced ANHEQ scales [61]. Any differences in ANHEQ scores were discussed to reach consensus [85,87,88]. In case any author was in charge of a study included in this scoping review, the remaining author(s) were decisive for the rating to minimize the risk of bias. If required, a fourth investigator (MSu) was consulted.

The ANHEQ scales were designed for Assessing Nordic Hamstring Exercise Quality of assessments and interventions specifically to evaluate the implementation and reporting of NHE-related evidence. These rating scales are more specifically applicable to NHE assessments and interventions than others, such as the TESTEX or PEDro scales [87,88]. The ANHEQ criteria comprise sensitive and reliable rating scales including eight items [61]. Their summated 13-point scores can be interpreted according to the American College Grading System: ‘excellent’ (13/12 points), ‘very good’ (11/10 points), ‘good’ (9/8 points), ‘average’ (7/6 points), ‘below average’ (5/4 points), ‘poor’ (3/2 points), ‘failure’ (1/0 points). Their use is recommended for sports scientists, physiotherapists, and coaches to verify the informative value of existing NHE-related evidence. A further rationale is to improve the design and reporting of future NHE studies and of NHE execution in everyday testing and training [61,85].

## 3. Results

### 3.1. Identification of Studies

The initial database search yielded a total of 194 potentially relevant studies. The reference lists’ inspection revealed 33 additional studies. After reviewing all abstracts and text bodies, 131 full-text articles were retrieved for final analyses; 71 NHE assessments (69 studies) and 83 NHE interventions (74 studies) were included in the scoping review, whereas 12 out of 131 studies were assigned to both categories (Figure 1).

### 3.2. Descriptive Characteristics of the Included NHE Assessments and Interventions

The specific characteristics of the 71 NHE assessments and 83 NHE interventions included in the scoping review are summarized in the Supplementary Tables S1 and S2 of the supplementary file; 72% of the analyzed assessments examined males, 20% both sexes and 6% females (3% not specified), whereas 81% of the interventions implemented males, 10% both sexes and 9% females. Samples of <20 and 20–50 participants were predominant for both assessments (45% and 31%) and interventions (65% and 15%). NHE assessments and interventions were executed mainly in team sports (65% and 78%), whereas 13% and 4% of the investigations did not specify the sports background of their participants.

**Figure 2:**
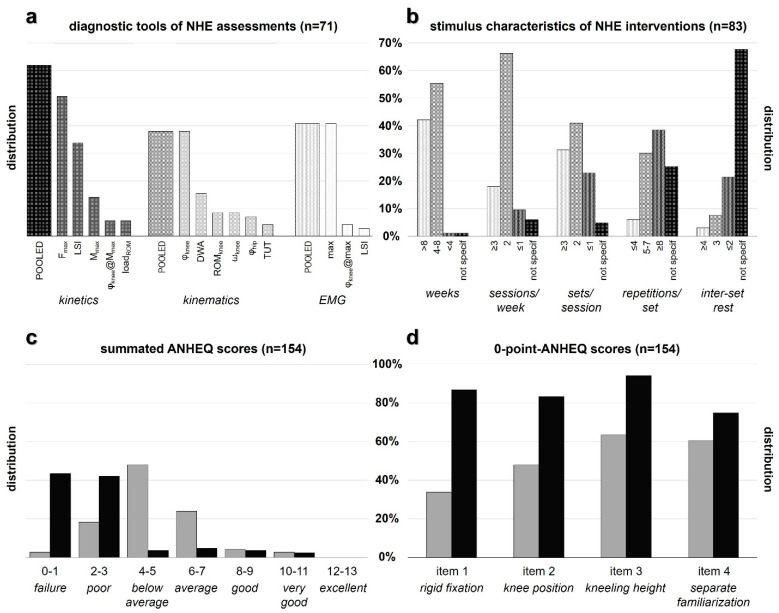
Percentage distributions of (**a**) diagnostic tools and parameters of NHE assessments (pooled according to the corresponding biomechanical measuring procedure) and (**b**) stimulus characteristics of NHE interventions. Total ANHEQ scores (**c**) and 0-point-ANHEQ scores (**d**) are presented for shared ANHEQ items 1–4 of assessments (grey bars) and interventions (black bars). *Footnote (abbreviations): NHE, Nordic Hamstring Exercise; F_max_, peak force; LSI, limb symmetry index; M_max_, peak moment;* *φ_knee_, knee angle; load_ROM_, load across entire range of motion; DWA, downward acceleration; ROM_knee_, knee range of motion;* *ω_knee_, mean knee angular velocity; φ_hip_, hip angle;* *TUT, time under tension; EMG, electromyography;* *not specif, not specified; ANHEQ, Assessing Nordic Hamstring Exercise Quality*.

NHE assessments usually analyzed force (51%), muscle activation (41%), knee angle kinematics (38%), and bilateral symmetry (37%). The average movement speed (8%), hip flexion (7%), the load across the entire ROM (6%), and the time under tension (4%) received less attention (Figure 2a). Typical NHE interventions implied a duration of 4–8 weeks (56%) and an exercise volume of 2 sessions per week (66%) with two sets per session (41%) and ≥8 repetitions per set (39%). Predominantly, the inter-set rest of multi-set interventions has not been specified (68%) (Figure 2b).

### 3.3. Total ANHEQ Scores

The total ANHEQ scores of the analyzed NHE assessments and interventions were 5.0 ± 2.0 and 2.0 ± 2.0 (median ± interquartile range), respectively (Supplementary Tables S1 and S2). The largest proportion of assessments was rated as ‘below average’ (49%) (Figure 2c). 42% and 43% of the interventions received the grading ‘poor’ and ‘failure’, respectively. A fraction of 7% of NHE assessments and 6% of NHE interventions were rated as ‘good’ and ‘very good’, whereas ‘excellent’ ANHEQ scores could not be assigned to any study. The majority of studies were assigned to ratings in the range from ‘average’ to ‘failure’ (Figure 2c).

### 3.4. Single ANHEQ Criteria Ratings

The largest proportions of 0-point-scores became apparent for consequences of impaired technique (87% for assessments) and kneeling height (94% for interventions) (Supplementary Tables S1 and S2). 0-point-scores were generally higher for interventions compared to assessments for rigid fixation (87% vs. 34%), knee position (83% vs. 48%), kneeling height (94% vs. 63%), and separate familiarization (75% vs. 61%) (Figure 2d). The ANHEQ criteria which received the highest possible score most frequently were rigid fixation (66% of assessments) and compliance (33% of interventions).

## 4. Discussion

### 4.1. Nordic Hamstring Exercise Quality

Based on the findings of this scoping review, the hypothesis that the quality of NHE assessments and interventions is ‘average’ and ‘below average’ must be rejected. ANHEQ ratings revealed that the quality of the 71 assessments (5.0 ± 2.0) and 83 interventions (2.0 ± 2.0) was generally ‘below average’ or rather ‘poor’ (Supplementary Tables S1 and S2, Figure 2c).

Overall, 34% of NHE assessments and 87% of NHE interventions applied the NHE with a partner (Supplementary Tables S1 and S2, Figure 2d), which might have impaired the execution quality and intensity compared to an execution with rigid fixation [12,61,65]. Also, 69% of NHE assessments and 94% of NHE interventions chose an inappropriately low kneeling height, which might have restricted the ROM at extended knee angles. Therefore, most studies did not implement optimal NHE modalities to assess strength capacities at relatively long muscle lengths or to elicit neuromuscular adaptations at the preventive target zone [35,48,49,50,61]. Further, 61% of NHE assessments and 75% of NHE interventions did not conduct a separate familiarization session so that the analyzed capacities might have been blended by the presence of a learning effect rather than reflecting the best possible performance (Figure 2d) [44].

The quality of NHE assessments was impaired by deficient or missing information about feedback of target movement speed (86%) and consequences of impaired technique (87%) (Supplementary Table S1). The load across the entire ROM (6%) and the time under tension (4%) were neglected by most NHE assessments (Figure 2a), although these parameters provide important information about the strength capacities and sustained muscle activation throughout the entire ROM [6,7,70,89]. NHE interventions demonstrated a high proportion of 0-point-scores for feedback of execution quality (88%) and inter-set rest (90%) (Supplement Table S2). Combined with the latter parameter, the predominantly high exercise volumes implemented during common interventions (39% with ≥8 repetitions/set) might have impaired the expedient development of maximal strength (Figure 2b) [61].

### 4.2. Limitations of Included Studies

Both NHE assessments and interventions suffered from imprecise reporting or lacking information regarding NHE execution modalities and subsequent analyses [61,85]. If the included full-text articles provided insufficient details (e.g., degree of cushioning for the rating of knee position), we rated the given information based on our interpretation. Most NHE studies instructed their participants to maintain a controlled forward-falling motion of their body for as long as possible [12,13,32,37,39,41,90,91]. We recommend that the NHE should not be associated with a fall or an uncontrolled descent but should rather be instructed as a controlled downward movement of the trunk, which emphasizes a sustained muscle activation at the end of the exercise. Therefore, practitioners and scientists should always stay alert to how their participants perform the NHE. If they do not perform the exercise with maximal effort and simply let their torsos fall down at the emergence of discomfort, the performance and subsequent adaptations will be compromised [39].

Overall, 66% of NHE assessments provided a deficient or missing presentation of their analyzed NHE variables so that their results can hardly be interpreted (Supplementary Table S1). Additionally, large standard deviations indicate substantial inter-individual differences in NHE execution quality and performance [6,7,41,72]. The generally poor study quality of current NHE interventions might be explained by the fact that 39% of the analyzed NHE interventions implemented the exercise as obligatory or optional part of a multi-exercise regimen [62]. However, the full potential of such complex training programs will only be realized if each single exercise is regularly adopted, correctly executed, progressed, and sustained by their intended end users [39,92,93,94]. Further, 22% of the included NHE interventions [5,11,13,25,28,29,31,34,36,37,43,54,55,56,57,58,59,60] implemented the intervention protocol of Mjølsnes et al. published in 2004 [12] (Supplement Table S2), although it is doubtful whether progression via exercise volume is an expedient means to elicit the largest adaptations [7,8,64]. Due to the high amount of additional stress and accumulated fatigue which is induced by this strenuous exercise, regular NHE exposure with unnecessarily high exercise volumes—39% of NHE interventions conducted ≥8 repetitions/set (Figure 2b)—might potentially have a negative rather than a positive effect on injury risk mitigation and performance [42].

Furthermore, most studies reported intended intervention regimens rather than presenting the actually performed exercise volumes. This deficiency might mask high inter-individual variability of exercise stimuli leading to divergent adaptations [94,95,96]. Only three studies reported data of NHE performances with high eccentric control beyond knee flexion angles of <45° [6,40,84]. Two recent studies which investigated NHE with 90° hip flexion only included participants who were able to perform more than 50% of the NHE knee ROM in a controlled manner [18,77]. Based on these small numbers, it can be assumed that most of the included NHE studies analyzed the performance and effects of the NHE, which involved a premature ending of the controlled eccentric action (<40° from the vertical) and a significant decrease in muscle activation [41,43,46,71,82,97,98,99]. It is a major challenge for future studies to overcome this major limitation of current NHE studies [11], because extended knee angles (~30° to 0° knee flexion) represent a target zone for injury prevention and rehabilitation [35,48,49,50].

### 4.3. Practical Recommendations

**Figure 3:**
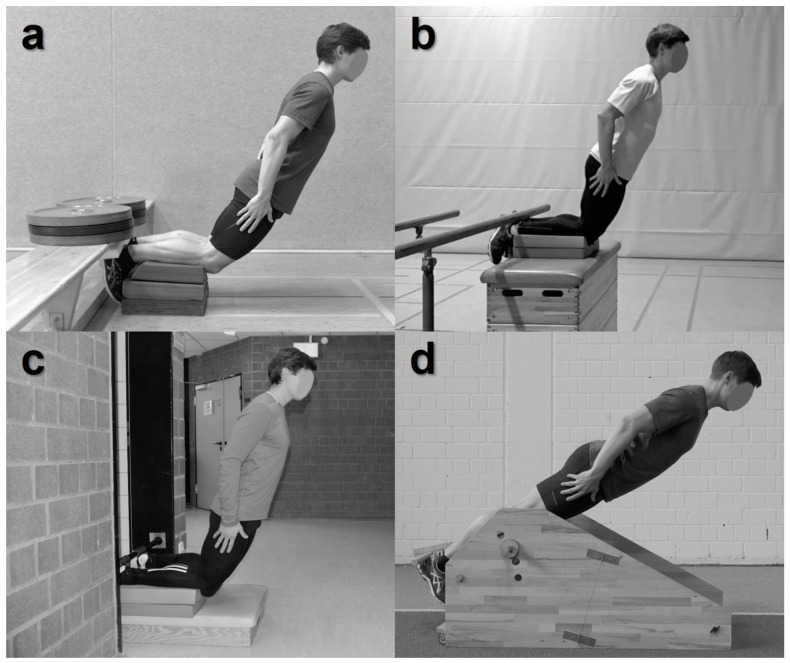
Representative Nordic Hamstring Exercise (NHE) training setups which provide fixed resistance to the heels, reasonable knee position, and expedient kneeling height: (**a**) appropriately weighted gym bench with cushion at the heels and at the shins (e.g., foam pads), (**b**) appropriately weighted bar, (**c**) doorway pull-up bar, and (**d**) a custom-made NHE device with modifiable shank inclination.

Previous conclusions of NHE-related research were unintentionally misinformed by recommendations based on potentially biased interpretations. Because of the lack of established and well-defined guidelines limited study and reporting quality are predominant (Figure 2c) [61,62]. Based on the insights of this scoping review, we subsequently aggregated and derived practical recommendations for NHE assessments and interventions (Table 1). These practical guidelines should contribute to improvements in the design and reporting of future NHE studies [61,85]. To enable the reproduction of NHE testing and everyday training settings and modalities, we encourage authors to provide informative and detailed images and/or sketches of their experimental setup (Figure 3). Supplementary video material showing exemplary NHE execution is appreciated.

#### 4.3.1. General Recommendations for NHE Execution Modality (ANHEQ Items 1–4)

We strongly recommend using a rigid fixation for safe and high-intensity NHE execution because a fixed resistance at the heels allows controlling the movement until full knee extension [1,12,61]. This is of crucial importance because injury prevention and rehabilitation programs should promote highest possible muscle activation throughout the entire ROM [43,46,55,100,101]. In contrast, the partner’s limited capacity to stabilize might cause an insufficient counterbalance that could impair the athlete’s ability to maximally perform the exercise and could limit the benefits of the NHE [12,65]. Mjølsnes et al. [12] emphasized that with increasing knee flexor strength, two or even three individuals will be needed to provide adequate support if the athlete reaches extended knee angles [12]. Consequently, the use of specialized equipment [44,48,66,68,71,80] or at least any stationary horizontal object [61,68] should be considered for NHE assessments and interventions (Figure 3).

To enable a physiological patella glide through the patellofemoral grove during the NHE, the shank’s support should reach the tuberositas tibiae. The knee joint should be free and should not touch the ground throughout the entire movement [61]. Practitioners and scientists should use foam pads, towel rolls, or related tools to place the knee joint on an edge and to provide appropriate cushioning to the shins [12,59,60,61,93]. The shanks should be placed at least 15 cm above the level that chest and/or hands touch at full knee extension [8,23,38,61,102,103] to accentuate the eccentric stimulus at extended muscle lengths. The particular knee flexion angles of ~30° to 0° mirror the critical ROM of hamstring injuries [35,104] and have the potential for biggest NHE-induced strength gains [56].

Although participants might be familiar with NHE execution, a separate and active familiarization to NHE procedures (e.g., fixation, knee position, kneeling height, target movement speed, feedback) is wanted 3 to 7 days prior to the assessment or the start of the intervention [61]. It should include a gradual approach to proper execution technique. Facilitations such as assistance (e.g., by elastic bands) [6,48,53,76] or shank inclination [18,44,75,77] might be implemented to convey the feeling for the constantly high muscle activation and to facilitate the control of the gravity-induced and progressively increasing overload during the last part of the ROM [55,101,105]. Thorough familiarization will improve the quality, reliability, and consistency of NHE performance [66,68,70,106,107,108] and consequently, the detection of players at elevated injury risk [109,110,111,112,113].

#### 4.3.2. Specific Recommendations for NHE Assessments (ANHEQ Items 5–8)

NHE assessments should present meaningful and comprehensive kinematic and kinetic data of supramaximal NHE performance [61]. It does not suffice to state that participants were instructed to oppose the increasing gravity-induced acceleration of their trunk for as long as possible by using their posterior thigh muscles as it is previously reported [12,13,24,32,37,39,41,90,114]. Instead, a quantification of how well participants fulfilled this task should be realized [40,41,43,71,82]. Optimally, unilateral load distribution across the entire ROM (e.g., impulse or work derived from camera-based analyses) should be presented in conjunction with the ROM to excessive downward acceleration. Continuous feedback of target movement speed is important to intensify the eccentric stimulus at extended muscle lengths where peak forces/moments are generated [6,42,45,51,102,106]. Variations of movement speed will affect muscle–tendon stiffness and muscle activation and thus force generation during execution [7,57,68,70,97,114]. NHE assessments that implement target velocities indicated as average cadence (e.g., 3 s or 30°/s) [41,42,43] should prevent muscle activation near full knee extension from becoming deficient. In order to improve the interpretation of the respective results, criteria of impaired technique (e.g., inadequate movement speed or excessive lumbar lordosis) and consequences should be explicitly defined (e.g., repeated or excluded from analysis) [66,68,70,81,106,115].

#### 4.3.3. Specific Recommendations for NHE Interventions (ANHEQ Items 5–8)

In contrast to most NHE interventions—only 4% applied ≤4 repetitions per set (Figure 2b), which implied a progression of exercise volume—emphasis should be put on exercise quality and intensity (e.g., a maximum of 4–6 repetitions per set) [6,8,39,53,64,94]. The goal of supramaximal NHE training is to enlarge the ‘muscle’s safe operating range’ [53]. Consequently, the accumulation of excessive inter-set fatigue-induced decreasing strength should be reduced because it will affect the muscle activation and will thus diminish the controlled ROM [42]. High intensity of effort and sustained muscle activation in the second half of the NHE will promote sarcomerogenesis, the mechanism by which eccentric training is thought to alter the muscle’s length–tension relationship due to the longitudinal addition of in-series sarcomeres [20,21,22,23,39,45,57,116].

We encourage practitioners and scientists to apply facilitations (e.g., increased shank inclination, assistance, hip flexion) to account for individualization and progression [6,44,48,53,75,76] as well as to delay the premature ending of the controlled action during the NHE [35,48,49,50]. Summed impulses or average time under tension can help to understand the inter-individual progress throughout the intervention [6,7]. Although this procedure is admittedly hard to realize in the field, individual thresholds to terminate an exercise set (e.g., ROM to downward acceleration <30°) should be implemented rather than predefining a fixed exercise volume. To improve the acute or chronic performance and quality of NHE execution by elevated motivation and effort [39,117], the use of visual and verbal feedback is recommended [6,42,106,117]. In cases where the optimal 1:1 coach-to-athlete ratio is not feasible (e.g., in larger samples), the athletes might give feedback to their training partner.

Since the high demands of the NHE are well-documented [6,41,42,43,44,45], an adequate inter-set rest period of ≥3 min, given an inter-repetition rest of at least 6 s between eccentric NHE repetitions [6,30,43,65,118], is required to develop maximal strength [6,22,23,30,42,61,65]. To elicit the desired neuromuscular adaptations, a high compliance with the intended NHE intervention program is needed [10,56,59,80,92,118,119,120]. The commonly low compliance might be improved by higher training intensity and quality [6,8,19,53,64] via reduced intervention volumes and by implementing facilitations that contribute to a constant training progress.

### 4.4. Implications & Perspectives

Although the great benefits of NHE testing and regular NHE training are well-known [2,3,83,103,104], the present scoping review revealed a ‘below average’ and ‘poor’ quality of published NHE assessments and interventions. These findings confirmed the current scoping review of Breed et al. [85], who demonstrated a predominantly low exercise intervention reporting quality of hamstring-related research. The NHE should no longer be introduced as partner exercise, which is easy to use because no additional equipment is required [3,11,13,15,20,121]. Instead, we recommend to emphasize its physically very demanding and fatiguing nature and the necessity to implement a rigid fixation at the heels [12,65], a reasonable knee position, and expedient kneeling height [61]. By meeting these requirements, progressively increasing gravitational moments at longer muscle lengths [40,47] can be withstood in a safe and appropriately comfortable way.

Further scientific evidence is needed to determine the optimal NHE training frequency [7,122] and periodized progression [6,7,30,39] as well as the timing within training sessions [56,58,81,123] and weeks [102]. It remains to be investigated if high-quality and high-intensity NHE execution will result in more reliable data, higher compliance, and greater neuromuscular adaptations—especially at longer muscle lengths—compared to traditional high-volume interventions [2,7,8,19,64,81]. Progression of exercise volume with increasing strength level, as suggested by the FIFA 11+ program [15,33,92,96,120,124,125,126,127,128,129,130,131,132,133,134], might not be the method of choice for purposeful injury prevention [94]. Furthermore, the implementation of single- or multiple-set NHE regimen as part of the warm-up should be reconsidered and should instead be performed during the cool-down routine [42,56,58,81,135].

Additional insights about the positive effects of increased shank inclination are required [18,44,75,77], especially for beginners and young, injured or weaker individuals [27,33,49,57,95,119,121,136,137,138,139]. Variations of the hip flexion angle, ROM, movement speed, and exercise intensity by externally provided assistance should be implemented in NHE interventions to gradually progress the intensity and load over time [6,18,48,53,57,76,77,97]. By decreasing the lever arm between the center of mass and the knee joint, a higher degree of hip flexion will reduce the maximal load posed on the knee flexors, which is required to perform an NHE. However, recent research has demonstrated the opposite, potentially because their participants were not strong enough to reach extended knee angles [44,48]. The effects of a physiological patellar glide (feasible vs. non-feasible) [61], the position of force application (calcaneus vs. calf) [83], and different rest periods on NHE performance, quality, and subsequent adaptations have not been investigated yet. So far, studies with strong participants who can control the movement until the second half of the exercise are rare [6,40]. Thus, the comparison between the effectiveness of the NHE compared to other resistance training exercises that address the knee flexors, such as the leg curl [12,38,140,141,142], hip extension [23,36,38,65,140], ’Romanian’ deadlifts [35,46,64,102,140,143], and ‘Russian’ belt [37,89], appeared to rely on different execution qualities of the analyzed exercises (e.g., due to their different physical and coordinative demands). If the participants possess the strength capacities to perform the NHE across the entire ROM—acknowledging that besides the hamstrings, other muscles (e.g., gastrocnemius, sartorius and gracilis) also contribute to eccentric knee flexor strength [44,45,70,77,100]—then the selective activation of the hamstring muscles (biceps femoris vs. semitendinosus) can be quantified with sufficient validity [48,55,81,101,105,140,144], (initial) movement speed can be increased [12,60,70,114], additional weight can be added [7,8,53,72], or even unilateral NHE can be executed [48,66,145]. Future research should focus on high NHE execution quality and intensity with the purpose of introducing a methodological exercise progression and periodization for all levels of expertise [39,47,49,72,94,146]. It should describe in detail which exercises should be performed with young, inexperienced, weak and/or injured individuals to learn a correct NHE across the entire ROM and how it can be modified and intensified [28,34,40,44,101,136]. If the physically very demanding NHE can be controlled until extended knee angles (~30° to 0° knee flexion), its characteristics appear to be very specific by mirroring the typical injury situation: an eccentric muscle action which elicits high muscle activation and high forces at nearly full knee extension (~600 to 750 N per leg) [12,22,35,72,114,145].

### 4.5. Limitations

This scoping review is restricted as the selection of studies was limited to the English language. For the search strategy, the authors used three wide databases. The number of databases could have been increased, but it can be assumed that the current state of research is well-represented by the large databases.The survey was carried out until December 2020 (e-pub date). This is due to the publication of the ANHEQ criteria [61]. The authors wanted to quantify the current state of NHE-related research prior to the recommendations presented in the ANHEQ framework.

## 5. Conclusions

This scoping review analyzed 131 full-text articles and demonstrated that the quality of NHE assessments and interventions was predominantly ‘below average’ or rather ‘poor’. Based on the current literature, we aggregated and derived practical recommendations for NHE assessments and interventions. These guidelines might contribute to improve the design and reporting of future NHE studies as well as of NHE execution in everyday testing and training. The full potential of the NHE can only be revealed if studies properly document and comply with high quality. Practitioners and scientists are encouraged to provide detailed information about their NHE modalities and about how their participants performed the exercise. Due to their different physical and coordinative demands, the effects of NHE reported in previous studies appeared to rely on heterogenous and diverging execution qualities. The appropriate setup of rigid fixation, reasonable knee position, expedient kneeling height, and thorough familiarization is suggested to be essential for best possible NHE performance and neuromuscular adaptations. NHE assessments should present comprehensive kinematic and kinetic data of supramaximal NHE performance, whereas NHE interventions should focus on exercise intensity and the implementation of facilitations. Future studies should overcome the revealed limitations of current NHE-related evidence by applying the ANHEQ criteria and the presented practical recommendations.

## Figures and Tables

**Table 1:** Practical recommendations for Nordic Hamstring Exercise (NHE) assessments and interventions following the criteria for Assessing Nordic Hamstring Exercise Quality (ANHEQ) [61].

**ANHEQ Criteria 1–4**	**NHE Assessments**	**NHE Interventions**	**Specific Comments**	**General Comments**
**Rigid fixation** *(item 1)*	devices which measure force or moment (in best case unilaterally)	wall bars, doorway pull-up bars, or any other solid and rigid horizontal object	use the calcanei as attachment point (rather than the Achilles tendon or the distal shank)	authors are encouraged to provide detailed and informative images and/or sketches of their experimental setup (Figure 3); supplementary video material showing exemplary NHE execution is appreciated
**Knee position** *(item 2)*	use foam pads, towel rolls, or related tools to place the knee joint on an edge and to provide appropriate cushion to the shins		knees should be free (support until tuberositas tibiae) and should not touch the ground	
**Kneeling height** *(item 3)*	provided that the shanks are horizontally aligned, they should be placed at least 15 cm above the floor (area which the chest and/or hands touch at full knee extension) to enable full knee extension		matches the approximate height of two foam pads	
**Separate familiarization** *(item 4)*	3 to 7 days prior to assessment or start of intervention participants become familiar with execution procedures; includes precise instructions, but above all a gradual approach to proper exercise execution technique; facilitations such as assistance (e.g., partner or elastic bands) or reduced range of motion should be used to convey the feeling for the correct execution technique		execute 2 sets of 3 repetitions from ~90° to 60° knee flexion followed by 3 sets of 3 assisted or guided repetitions across the full range of motion (~90° to 0°)	authors should report in sufficient detail when and how familiarization took place and how experienced the participants are with regard to the NHE
**ANHEQ criteria 5–8**	**NHE Assessments**		**Specific Comments**	**General Comments**
**Diagnostic tools** (item 5)	in best case, kinematic (e.g., range of motion to downward acceleration) and kinetic data (e.g., peak force/moment) are analyzed/captured		ensure that force transducers are in line with the direction of force application; if possible, calculate joint moments by using the camera-based position of the knee joint	authors are encouraged to present data of single or multiple exemplary NHE repetitions
**Feedback of target movement speed** (item 6)	continuous angle-time information is provided (e.g., via webcam) in real time to the participants by a monitor		cross-fade the sagittal real time video with a stick figure moving at target movement speed	authors are supposed to add respective sentences to the method section dealing with these issues or they should provide supplementary material
**Consequences of impaired technique** (item 7)	criteria of impaired technique (e.g., inadequate movement speed or excessive lumbar lordosis) and consequences are explicitly defined (e.g., repeated or excluded from analysis)		admittedly not entirely selective, but the indication is important to evaluate the results	
**Presentation of NHE performance variables** (item 8)	appropriate tables and/or figures illustrate representative or average data, which quantify NHE performance		e.g., force-time or moment-angle history, range of motion to downward acceleration, time under tension	authors should present relevant parameters either in the main body or as supplementary material
**ANHEQ criteria 5–8**	**NHE Interventions**		**Specific Comments**	**General Comments**
**Progression & individualization of program variables** (item 5)	exercise intensity and/or volume of eccentric-only NHE training progress and inter-individual differences are respected and/or assessed (e.g., summed impulses)		focus on exercise quality and intensity (e.g., a maximum of 6 repetitions per set); consider applying facilitations (e.g., increased shank inclination, assistance, hip flexion) to account for individualization and progression)	authors are encouraged to implement individual thresholds to terminate an exercise set (e.g., range of motion to downward acceleration <30°) rather than predefining a fixed exercise volume
**Feedback of execution quality** (item 6)	in best case, a combined real time feedback is provided which includes visual (e.g., on a monitor) and audible (e.g., by a coach or physiotherapist) information in a 1:1 coach-to-athlete ratio		admittedly not possible in large samples; alternatively the athletes might give feedback to their training partner	authors should report in sufficient detail how and which feedback is given
**Inter-set rest** (item 7)	an adequate rest period of ≥3 min (given an inter-repetition rest of ≥6 s between eccentric NHE) counteracts the accumulation of excessive muscular fatigue across sets and ensures the preservation of both exercise quality and intensity		pool athletes of similar strength capacities together (e.g., 3 athletes form a training group); while the first athlete exercises, the second provides assistance and the third one gives feedback; in this form, 3 min rest are reasonably filled	authors are supposed to add a respective sentence to the method section
**Compliance** (item 8)	in best case, all participants performed ≥85% of the intended NHE repetitions/sets with a low inter-individual variability of compliance		refer more specifically to compliance with NHE repetitions/sets rather than training sessions because repetitions/sets per session might differ	authors should indicate a mean value of NHE-specific compliance, but in case of certain variability, an inter-individual range should be reported as well

## Data Availability

Full dataset and/or statistical code are available from the corresponding author.

## Supplementary Materials

The following supporting information can be downloaded at: https://www.mdpi.com/article/10.3390/ijerph191811225/s1, Table S1. Characteristics and methodological details as well as ANHEQ scores of the 71 included NHE assessments (69 studies). Table S2. Characteristics and methodological details as well as ANHEQ scores of the 83 included NHE interventions (74 studies).
