# Supplementary material for: Quo Vadis Nordic Hamstring Exercise-Related Research?—A Scoping Review Revealing the Need for Improved Methodology and Reporting
Source: Int J Environ Res Public Health. 2022 Sep 7;19(18):11225. doi: 10.3390/ijerph191811225 (PMC9517005; doi:10.3390/ijerph191811225)
Supplement: Supplementary file 1 [file ijerph-19-11225-s001.zip › ijerph-1851671-supplementary.pdf]

Table S1. Characteristics and methodological details as well as ANHEQ scores of the 71 included NHE assessments (69 studies).

**study**	**sample**			**execution**		**analysis**		**ANHEQ assessment scores**								
	**sample size** analysed (recruited)	**sex**	**sports background**	**NHE modality**	**illustration** (detailed & informative)	**methods** (implemented & analysed)	**parameters** (analysed & presented)	**sum**	rigid fixation	knee position	kneeling height	familiarization	diagnostic tools	movement speed	impaired technique	NHE performance
**distribution**<br>2 points<br>1 point<br>0 points								**median**<br>**±IQR**<br>**5.0±2.0**	**66%**<br>**0%**<br>**34%**	**0%**<br>**52%**<br>**48%**	**0%**<br>**37%**<br>**63%**	**0%**<br>**39%**<br>**61%**	**31%**<br>**66%**<br>**3%**	**3%**<br>**11%**<br>**86%**	**0%**<br>**13%**<br>**87%**	**16%**<br>**18%**<br>**66%**
Alt et al. (2018) [6]	16	m	track & field (sprint)	dynamometer (padded, rope assistance)	4 pictures	dyna<br>MoCap (3D)	M_{max}<br>$load_{ROM}$ (J)<br>TUT<br>φ_{knee}<br>ROM_{knee}<br>ω_{knee}<br>ROM_{DWA}<br>φ_{hip}	**10**	2	1	0	1	2	2	0	2
Arnason et al. (2014) [116]	18	m & f	soccer	partner (folded mat)	3 pictures	EMG	EMG_{max}<br>LSI_{EMG}	**2**	0	1	0	0	1	0	0	0
Beuchat & Maffiuletti (2019) [90]	20	m & f	not specified	partner (mat)	1 picture (2 positions)	gonio<br>EMG	φ_{knee}<br>ROM_{knee}<br>EMG_{max}	**5**	0	1	0	0	2	0	0	2
	10	m	soccer	partner	N/A	gonio	φ_{knee}	**4**	0	0	0	0	2	1	0	1

Blandford et al. (2018) [104]						EMG	EMG_{max}									
Bourne et al. (2015) [102]	178 (194)	m	rugby	NordBord prototype (elevated, foam pad)	3 pictures	dyna	F_{max} LSI_{Fmax}	**5**	2	1	1	0	1	0	0	0
Bourne et al. (2017a) [38]	24	m & f	recreationally active	NordBord prototype (elevated, foam pad)	2 pictures	EMG	EMG_{max}	**6**	2	1	1	1	1	0	0	0
Bourne et al. (2019) [67]	84 (90)	f	Australian Rules football	NordBord (padded)	2 pictures	dyna	F_{max} LSI_{Fmax}	**5**	2	1	1	0	1	0	0	0
Buchheit et al. (2016) [106]	122	m	soccer, Australian Rules football	NordBord prototype (elevated)	4 pictures	dyna	F_{max}	**6**	2	0	1	0	1	0	0	2
Bueno et al. (2021) [74]	15 (20)	m	soccer	custom-made device (elevated)	1 picture	dyna	F_{max}	**6**	2	0	1	0	1	1	1	0
Burrows et al. (2020) [76]	16	m	soccer	custom-made frame (foam pad)	N/A	dyna<br>EMG	F_{max}<br>EMG_{max}	**5**	2	1	0	0	2	0	0	0
Chalker et al. (2016) [145]	74	m	cricket	NordBord prototype (foam pad)	N/A	dyna	F_{max} LSI_{Fmax}	**5**	2	1	0	1	1	0	0	0
Chalker et al. (2018) [105]	44	m	cricket	NordBord prototype (foam pad)	N/A	dyna	F_{max} LSI_{Fmax}	**6**	2	1	0	1	1	0	1	0
	24 (29)	m			N/A	dyna	F_{max}	**8**	2	0	0	1	2	0	1	2

Chesterton et al. (2021) [69]			recreationally active	Hamstring Solo Elite		MoCap (3D)<br>EMG	φ_{knee}<br>φ_{DWA}<br>EMG_{max}									
Comfort et al. (2017) [99]	15	m	soccer, rugby	partner (thin mat)	1 picture	EMG	EMG_{max}	**2**	0	0	0	1	1	0	0	0
Delahunt et al. (2016) [43]	15	m	recreationally active	partner	N/A	MoCap (2D)<br>EMG	φ_{knee}<br>ROM_{knee}<br>φ_{DWA}<br>EMG_{max}	**4**	0	0	0	0	2	0	0	2
de Oliveira et al. (2020) [73]	25	m	soccer	custom-made device (elevated)	1 picture	dyna	F_{max}	**4**	2	0	1	0	1	0	0	0
Ditroilo et al. (2013) [41]	18	m	field sports	partner	N/A	gonio<br>EMG	φ_{knee}<br>ROM_{knee}<br>φ_{DWA}<br>EMG_{max}<br>φ_{knee}@EMG_{max}	**4**	0	0	0	0	2	0	0	2
Drury et al. (2020) [135]	24	m	soccer	NordBord (padded)	N/A	dyna	F_{max}	**5**	2	1	1	0	1	0	0	0
Duhig et al. (2019) [22]	30	m	recreationally active	NordBord prototype (foam pad)	N/A	dyna	M_{max}	**4**	2	1	0	0	1	0	0	0
Ebben (2009) [78]	34	m & f	not specified	Glute-Ham machine (elevated)	N/A	EMG	EMG_{max}	**4**	2	0	1	0	1	0	0	0
Engebretsen et al. (2010) [17]	508 (769)	m	soccer	partner (thin mat)	2 pictures	N/A		**0**	0	0	0	0	0	0	0	0

Fernandez-Gonzalo et al. (2016) [89]	9	m	soccer	partner	N/A	gonio	φ_{knee}<br>ROM_{knee}<br>TUT	**2**	0	0	0	0	1	0	0	1
Franchi et al. (2019) [138]	170	m & f	alpine skiing	Nordbord (padded)	N/A	dyna	F_{max}<br>LSI_{Fmax}	**5**	2	1	1	0	1	0	0	0
Freeman et al. (2019) [27]	14	m & f	field sports	NordBord (padded)	N/A	dyna	F_{max}	**6**	2	1	1	1	1	0	0	0
Giakoumis et al. (2020) [107]	44	m & f	track & field (sprint, long jump, combined)	NordBord (padded)	2 pictures	dyna	M_{max}<br>LSI_{Mmax}<br>F_{max}<br>LSI_{Fmax}	**5**	2	1	1	0	1	0	0	0
Guruhan et al. (2020) [143]	31	m & f	not specified	unclear (partner, thin mat or belt)	2 pictures	EMG	EMG_{max}	**2**	0	0	0	1	1	0	0	0
Hegyi et al. (2018) [142]	12	m	recreationally active	NordBord prototype (foam pad)	N/A	dyna<br>EMG	F_{max}<br>EMG_{max}	**7**	2	1	0	1	2	1	0	0
Hegyi et al. (2019) [48]	13	not specified	soccer, rugby	Hamtech	4 pictures (4 variations)	dyna<br>potentiometry<br>EMG	M_{max}<br>F_{max}<br>φ_{knee}<br>EMG_{max}	**6**	2	0	0	1	2	1	0	0
Iga et al. (2012) [55]	10	m	soccer	partner (thin mat)	1 sketch (2 positions)	gonio<br>EMG	φ_{knee}<br>EMG_{max}<br>LSI_{EMG}	**5**	0	0	0	0	2	1	0	2
Isık et al. (2018) [137]	88	m	soccer	Nordbord (padded)	N/A	dyna	F_{max}<br>LSI_{Fmax}	**5**	2	1	1	0	1	0	0	0

Lacome et al. (2019) [64]	19	m	soccer	NordBord (padded)	N/A	dyna	F_{max}	**6**	2	1	1	1	1	0	0	0
Lee et al. (2017) [82]	30	m	soccer	partner (thin mat)	3 pictures	MoCap (2D)	φ_{knee}<br>φ_{DWA}	**2**	0	0	0	0	1	0	0	1
Lee et al. (2018) [71]	25	m	soccer	custom-made stabilization platform (elevated)	3 pictures	MoCap (2D)	φ_{knee}<br>φ_{DWA}	**5**	2	0	1	0	1	0	0	1
Lodge et al. (2020) [68]	19 (26)	m	field sports	Hamstring Solo Elite	1 picture	dyna	F_{max}<br>LSI_{Fmax}	**5**	2	0	0	1	1	0	1	0
Lovell et al. (2016) [81]	12	m	soccer	partner	1 sketch	gonio<br>EMG	φ_{knee}<br>ω_{knee}<br>EMG_{max}	**6**	0	0	0	1	2	1	1	1
Markovic et al. (2020) [136]	155	m	soccer	NordBord prototype	N/A	dyna	M_{max}<br>LSI_{Mmax}<br>F_{max}<br>LSI_{Fmax}	**3**	2	0	0	0	1	0	0	0
Marshall et al. (2015) [42]	10	m	soccer	partner	N/A	gonio<br>EMG	φ_{knee}<br>ω_{knee}<br>EMG_{max}	**6**	0	0	0	1	2	1	0	2
Marušič & Sarabon (2020) [77]	33	not specified	not specified	custom-made device (elevated)	1 picture	EMG	EMG_{max}	**4**	2	0	1	0	1	0	0	0
Marušič et al. (2020) [18]	18	m & f	recreationally active	custom-made device (elevated)	2 pictures	dyna	F_{max}	**4**	2	0	1	0	1	0	0	0
	33	m	rugby		N/A	dyna	F_{max}	**6**	2	1	1	0	2	0	0	0

McGrath et al. (2020) [144]				NordBord (padded)		MoCap (3D)	φ_{knee}<br>TUT									
Messer et al. (2020) [115]	14	m & f	recreationally active	NordBord (padded)	N/A	dyna	F_{max}<br>LSI_{Fmax}	**6**	2	1	1	1	1	0	0	0
Monajati et al. (2017) [100]	10	f	soccer	partner (thin mat)	4 pictures	MoCap (3D)<br>EMG	φ_{knee}<br>EMG_{max}	**5**	0	0	0	1	2	0	0	2
Narouei et al. (2018) [96]	10	m	not specified	partner	N/A	MoCap (3D)<br>EMG	φ_{knee}<br>φ_{DWA}<br>EMG_{max}	**4**	0	0	0	0	2	1	0	1
O'Boyle et al. (2021) [134]	10	m	soccer	partner	N/A	EMG	EMG_{max}	**2**	0	0	0	1	1	0	0	0
Opar et al. (2013) [66]	30	m	Australian Rules football, rugby, soccer, sprint	NordBord prototype (foam pad)	3 pictures	dyna	F_{max}<br>LSI_{Fmax}	**6**	2	1	0	1	1	0	1	0
Opar et al. (2015a) [83]	210	m	Australian Rules football	NordBord prototype (foam pad)	N/A	dyna	F_{max}<br>LSI_{Fmax}	**4**	2	1	0	0	1	0	0	0
Opar et al. (2015b) [112]	99	m	Australian Rules football	NordBord prototype (elevated, foam pad)	4 pictures	dyna	F_{max}<br>LSI_{Fmax}	**5**	2	1	1	0	1	0	0	0
Page et al. (2020) [97]	18	m	soccer	partner	N/A	MoCap (2D)	φ_{knee}<br>φ_{DWA}	**3**	0	0	0	1	1	0	0	1
Pedersen et al. (2020) [141]	15	m	soccer	partner	1 picture (thin mat)	EMG	EMG_{max}	**2**	0	0	0	1	1	0	0	0

Pollard et al. (2019) [8]	20	m	recreationally active	NordBord prototype (elevated, foam pad)	3 pictures	dyna	F_{max}<br>LSI_{Fmax}	**6**	2	1	1	1	1	0	0	0
Presland et al. (2018) [7]	20	m	recreationally active	NordBord prototype (foam pad)	N/A	dyna	F_{max}<br>LSI_{Fmax}<br>$load_{ROM}$ (J)	**5**	2	1	0	1	1	0	0	0
Ribeiro-Alvares et al. (2020) [111]	210	m	soccer	custom-made device (elevated)	1 picture	dyna	F_{max}<br>LSI_{Fmax}	**4**	2	0	1	0	1	0	0	0
Rossi et al. (2020) [75]	9	m	not specified	custom-made device (elevated)	1 picture	dyna<br>MoCap (3D)<br>EMG	F_{max}<br>LSI_{Fmax}<br>φ_{knee}<br>ROM_{knee}<br>ω_{knee}<br>EMG_{max}	**6**	2	0	1	1	2	0	0	0
Sadri-Aghdam et al. (2020) [84]	44	m	soccer, track & field	partner (padded mat)	3 pictures	MoCap (2D)	φ_{knee}<br>φ_{DWA}	**2**	0	1	0	0	1	0	0	0
Sarabon et al. (2019) [44] *Fig. 1A, D, F*	18	m & f	not specified	custom-made device (padded)	3 pictures (3 variations, 3 positions each)	dyna<br>MoCap (3D)<br>EMG	M_{max}<br>φ_{knee}@M_{max}<br>φ_{knee}<br>φ_{hip}<br>EMG_{max}	**7**	2	1	0	0	2	0	0	2
Sarabon et al. (2019) [44] *Fig. 1B, C, E*	18	m & f	not specified	custom-made device (padded)	4 pictures (3 variations, 1-3 positions each)	dyna<br>MoCap (3D)	M_{max}<br>φ_{knee}@M_{max}<br>φ_{knee}<br>φ_{hip}	**8**	2	1	1	0	2	0	0	2

						EMG	EMG_{max}									
Sconce et al. (2015) [40]	16	m & f	soccer	partner (thin mat)	3 pictures	MoCap (2D)	φ_{knee}<br>φ_{DWA}	**2**	0	0	0	0	1	0	0	1
Shalay et al. (2020) [16]	143	m	soccer	not specified	N/A	N/A	N/A	**0**	0	0	0	0	0	0	0	0
Suarez-Arrones et al. (2019) [26]	33	m	soccer	Acceleration LegCurl (padded)	N/A	dyna	F_{max}	**5**	2	1	0	1	1	0	0	0
Timmins et al. (2016a) [110]	15	m	soccer, Australian Rules football	NordBord prototype (foam pad)	N/A	dyna	F_{max}<br>LSI_{Fmax}	**4**	2	1	0	0	1	0	0	0
Timmins et al. (2016b) [103]	152	m	soccer	NordBord prototype (foam pad)	N/A	dyna	M_{max}<br>LSI_{Mmax}<br>F_{max}<br>LSI_{Fmax}	**4**	2	1	0	0	1	0	0	0
Tsaklis et al. (2015) [46]	20	f	track & field (sprint, jumps)	partner (folded mat)	1 picture	EMG	EMG_{max}	**2**	0	1	0	0	1	0	0	0
van den Tillaar et al. (2017) [72]	12	m	not specified	custom-made device (thin mat)	5 pictures	MoCap (3D)<br>EMG	φ_{knee}<br>φ_{hip}<br>EMG_{max}<br>φ_{knee}@EMG_{max}	**6**	2	0	0	1	2	0	0	1
van Dyk et al. (2017) [108]	413	m	soccer	NordBord prototype (foam pad)	N/A	dyna	F_{max}<br>LSI_{Fmax}	**4**	2	1	0	0	1	0	0	0
van Dyk et al. (2018) [109]	288	m	soccer	NordBord prototype (foam pad)	N/A	dyna	F_{max}<br>LSI_{Fmax}	**4**	2	1	0	0	1	0	0	0

Vercelli et al. (2020) [98]	5	m & f	recreationally active	partner (padded mat)	2 pictures	MoCap (2D)	$φ_{knee}$<br>$φ_{DWA}$	**3**	0	1	0	0	1	0	0	1
Vicens-Bordas et al. (2020) [114]	284 (306)	m	soccer	NordBord prototype (foam pad)	N/A	dyna	F_{max}<br>LSI_{Fmax}	**5**	2	1	0	0	1	0	1	0
Wiesinger et al. (2019) [70] *NHD₃₀*	25	m	recreationally active	custom-made device (elevated)	1 sketch	dyna<br>MoCap (2D)<br>EMG	M_{max}<br>$φ_{knee}@M_{max}$<br>LSI_{Mmax}<br>$load_{ROM}$ (W)<br>$φ_{knee}$<br>$ω_{knee}$<br>EMG_{max}	**10**	2	0	1	1	2	2	1	1
Wiesinger et al. (2019) [70] *NHD_{max}*	25	m	recreationally active	custom-made device (elevated)	1 sketch	dyna<br>MoCap (2D)<br>EMG	M_{max}<br>$φ_{knee}@M_{max}$<br>LSI_{Mmax}<br>$load_{ROM}$ (W)<br>$φ_{knee}$<br>$ω_{knee}$<br>EMG_{max}	**8**	2	0	1	1	2	0	1	1
Wik et al. (2018) [146]	159	m	soccer	Nordbord (padded)	N/A	dyna	F_{max}	**5**	2	1	1	0	1	0	0	0
Zebis et al. (2013) [139]	16	f	handball, soccer	partner (foam pad)	2 pictures	magnetic tracking<br>EMG	$φ_{knee}$<br>$φ_{hip}$<br>EMG_{max}<br>$φ_{knee}@EMG_{max}$	**5**	0	1	0	1	2	0	0	1

abbreviations: NHE, Nordic Hamstring Exercise; ANHEQ, Assessing Nordic Hamstring Exercise Quality; IQR, interquartile range; m, males; f, females; N/A, not available; dyna, dynamometry; MoCap (2D/3D), 2D/3D camera-based motion capture; gonio, goniometry; EMG, electromyography; M_{max}, peak moment; $load_{ROM}$ (J/W), load across entire ROM

quantified via impulse (J) or work (W); TUT, time under tension; φ_{knee}, knee angle; ROM_{knee}, knee range of motion; ω_{knee}, mean knee angular velocity; ROM_{DWA}, range of motion till downward acceleration; φ_{hip}, hip angle; LSI, limb symmetry index; F_{max}, peak force; φ_{DWA}, knee angle at downward acceleration; EMG_{max}, peak muscle activity.

Table S2. Characteristics and ANHEQ scores of the 83 included NHE interventions (74 studies).

**Study**	**sample**			**execution & intervention**			**ANHEQ intervention scores**								
	**sample size** analysed (recruited)	**sex**	**sports background**	**NHE modality**	**illustration** (detailed & informative)	**intervention volume** ∑ weeks<br>mean sessions/weeks (range)<br>mean sets/session (range)<br>mean repetitions/sets (range)	**sum**	rigid fixation	knee position	kneeling height	familiarization	progression	execution quality	interest rest	compliance
**distribution**<br>2 points<br>1 point<br>0 points							**median**<br>**±IQR**<br>**2.0±2.0**	**13%**<br>**0%**<br>**87%**	**0%**<br>**17%**<br>**83%**	**0%**<br>**6%**<br>**94%**	**0%**<br>**25%**<br>**75%**	**7%**<br>**56%**<br>**37%**	**2%**<br>**10%**<br>**88%**	**0%**<br>**10%**<br>**90%**	**33%**<br>**1%**<br>**66%**
Akbari et al. (2020) [128]	12	m	soccer	partner	N/A	8 weeks<br>3.0 (3)<br>1.0 (1)<br>3.0 - 15.0 (3 - 15)	**0**	0	0	0	0	0	0	0	0
Alonso-Fernandez et al. (2018a) [20]	23	m	recreationally active	partner (thin mat)	3 pictures	8 weeks<br>2.8 (2 - 3)<br>2.8 (2 - 3)<br>7.5 (4 - 10)	**3**	0	0	0	1	1	1	0	0
Alonso-Fernandez et al. (2018b) [21]	23	m	recreationally active	partner (thin mat)	3 pictures	8 weeks<br>2.8 (2 - 3)<br>2.8 (2 - 3)<br>7.5 (4 - 10)	**3**	0	0	0	1	1	1	0	0

Alt et al. (2018) [6]	16	m	track & field (sprint)	dynamometer (padded, rope assistance)	4 pictures	4 weeks<br>3.0 (3)<br>3.0 (3)<br>3.0 (3)	**10**	2	1	0	0	2	2	1	2
Alt et al. (2021) [30]	12	m	track & field (sprint)	dynamometer (padded, rope assistance)	2 pictures	4 weeks<br>3.0 (3)<br>3.0 (3)<br>3.0 (3)	**11**	2	1	0	1	2	2	1	2
Anastasi & Hamzeh (2011) [31]	13	f	rugby	partner (thin mat)	3 pictures	10 weeks<br>3.0 (3)<br>3.0 (3)<br>8.0 (6 - 10)	**1**	0	0	0	0	1	0	0	0
Arnason et al. (2008) [11]	not specified (30 teams with 18-24 players each)	m	soccer	partner (thin mat)	1 sketch (2 positions)	2 seasons<br>N/A (1 - 3)<br>N/A (2 - 3)<br>N/A (5 - 12)	**1**	0	0	0	0	1	0	0	0
Arsenis et al. (2020) [130]	16	m	soccer	partner	N/A	8 weeks<br>3.0 (3)<br>1.0 (1)<br>7.0 - 10.0 (7 - 10)	**1**	0	0	0	1	0	0	0	0
Bourne et al. (2017b) [23]	10	m	recreationally active	NordBord prototype (elevated, foam pad)	2 pictures	10 weeks<br>2.0 (2)<br>4.4 (2 - 6)<br>7.3 - 8.3 (5 - 10)	**9**	2	1	1	1	1	0	1	2
Brito et al. (2010) [123]	18 (20)	m	soccer	partner	N/A	10 weeks<br>3.0 (3)	**0**	0	0	0	0	0	0	0	0

						1.0 (1)<br>3.0 - 15.0 (3 - 15)									
Brooks et al. (2006) [63]	200	m	rugby	partner	N/A	2 seasons<br>1.3 (N/A)<br>2.8 (N/A)<br>6.7 (N/A)	**0**	0	0	0	0	0	0	0	0
Chaabene et al. (2020) [28]	10	f	handball	partner	N/A	8 weeks<br>2.6 (1 - 3)<br>2.9 (2 - 3)<br>8.4 - 9.3 (5 - 12)	**3**	0	0	0	0	1	0	0	2
Clark et al. (2005) [32]	9	m	Australian Rules football	partner (thin towel)	2 pictures	4 weeks<br>2.3 (1 - 3)<br>2.7 (2 - 3)<br>6.7 (5 - 8)	**1**	0	0	0	0	1	0	0	0
Daneshjoo et al. (2013) [124]	12	m	soccer	partner	N/A	8 weeks<br>3.0 (3)<br>1.0 (1)<br>3.0 - 15.0 (3 - 15)	**0**	0	0	0	0	0	0	0	0
Delahunt et al. (2016) [43]	15	m	recreationally active	partner	N/A	6 weeks<br>2.5 (1 - 3)<br>2.8 (2 - 3)<br>8.1 (5 - 12)	**2**	0	0	0	0	1	0	1	0
del Ama Espinosa et al. (2015) [14]	22 (24)	f	soccer	not specified	N/A	21 weeks<br>2.0 (2)<br>1.0 (1)<br>5.0 (5)	**1**	0	0	0	0	0	0	0	1
Delextrat et al. (2020) [120]	9 (10)	f	field hockey	partner (thin mat)	2 pictures	6 weeks<br>3.0 (3)	**1**	0	0	0	0	1	0	0	0

						2.6 (2 - 3)<br>8.0 (6 - 10)									
de Oliveira et al. (2020) [73]	25	m	soccer	partner	N/A	4 weeks<br>2.0 (2)<br>3.0 (3)<br>8.0 - 8.5 (6 - 10)	**3**	0	0	0	0	1	0	0	2
Drury et al. (2020) [135]	24	m	soccer	partner	N/A	6 weeks<br>1.8 (1 - 2)<br>2.5 (2 - 3)<br>6.0 (5 - 8)	**3**	0	0	0	0	1	0	0	2
Duhig et al. (2019) [22]	15	m	recreationally active	NordBord prototype (foam pad)	N/A	5 weeks<br>1.8 (1 - 2)<br>3.7 (2 - 5)<br>6.0 (6)	**8**	2	1	0	0	2	0	1	2
Elerian et al. (2019) [122] *pre-training*	16 (17)	m	soccer	partner	2 pictures	12 weeks<br>1.9 (1 - 2)<br>3.7 (2 - 4)<br>8.0 (5 - 12)	**3**	0	0	0	0	1	0	0	2
Elerian et al. (2019) [122] *pre- & post-training*	16 (17)	m	soccer	partner	2 pictures	12 weeks<br>3.8 (2 - 4)<br>2.8 (3 - 6)<br>6.6 (2 - 12)	**3**	0	0	0	0	1	0	0	2
Engebretsen et al. (2008) [59]	76	m	soccer	partner (thin mat)	2 pictures	10 weeks<br>2.7 (1 - 3)<br>2.9 (2 - 3)<br>9.2 (5 - 12)	**1**	0	0	0	0	1	0	0	0
Freeman et al. (2019) [27]	14	m & f	field sports	not specified	N/A	4 weeks<br>2.0 (2)	**5**	0	0	0	1	1	0	1	2

						2.8 (2 - 3)<br>5.0 (4 - 6)									
Gabbe et al. (2006) [117]	114	m	Australian Rules football	partner (thin towel)	3 pictures	12 weeks<br>0.4 (0 - 1)<br>12.0 (12)<br>6.0 (6)	**1**	0	0	0	0	0	0	1	0
Grooms et al. (2013) [125]	34	m	soccer	partner	N/A	12 weeks<br>N/A (5 - 6)<br>1.0 (1)<br>5.0 - 15.0 (5 - 15)	**0**	0	0	0	0	0	0	0	0
Harøy et al. (2017) [126]	16 (23)	m	soccer	partner	N/A	8 weeks<br>3.0 (3)<br>1.0 (1)<br>3.0 - 15.0 (3 - 15)	**2**	0	0	0	0	0	0	0	2
Hasebe et al. (2020) [54]	156	m	soccer	partner	1 sketch (2 positions)	27 weeks<br>2.0 (1 - 2)<br>2.9 (2 - 3)<br>8.2 (5 - 10)	**3**	0	0	0	0	1	0	0	2
Iga et al. (2012) [55]	10	m	soccer	partner (thin mat)	1 sketch (2 positions)	4 weeks<br>2.3 (1 - 3)<br>2.7 (2 - 3)<br>6.7 (5 - 8)	**1**	0	0	0	0	1	0	0	0
Ishøi et al. (2018) [25]	11 (18)	m	soccer	partner	N/A	10 weeks<br>2.7 (1 - 3)<br>2.9 (2 - 3)<br>9.0 - 9.4 (5 - 12)	**1**	0	0	0	0	1	0	0	0
Jakobsen et al. (2017) [79]	5	m & f	not specified	step-bench	N/A	4 weeks<br>3.0 (3)	**2**	2	0	0	0	0	0	0	0

						3.0 (3)<br>6.0 - 8.0 (6 - 8)									
Krommes et al. (2017) [29]	9	m	soccer	partner	N/A	10 weeks<br>2.7 (1 - 3)<br>2.9 (2 - 3)<br>9.0 - 9.4 (5 - 12)	**3**	0	0	0	0	1	0	0	2
Lacome et al. (2019) [64] *low volume*	19	m	soccer	partner (foam pad)	2 pictures<br>1 sketch	6 weeks<br>1.0 (1)<br>1.0 (1)<br>4.0 (4)	**2**	0	1	0	1	0	0	0	0
Lacome et al. (2019) [64] *high volume*	19	m	soccer	partner (foam pad)	2 pictures<br>1 sketch	6 weeks<br>1.0 (1)<br>4.0 (4)<br>4.0 (4)	**2**	0	1	0	1	0	0	0	0
Lopes et al. (2020) [129]	31 (37)	m	futsal	partner	N/A	20 weeks<br>1.8 (1 - 2)<br>0.9 (1)<br>3.0 - 15.0 (3 - 15)	**0**	0	0	0	0	0	0	0	0
Lovell et al. (2018a) [56]	24 (30)	m	soccer	partner	N/A	12 weeks<br>1.9 (1 - 2)<br>3.7 (2 - 4)<br>8.0 (5 - 12)	**2**	0	0	0	1	1	0	0	0
Lovell et al. (2018b) [101]	18	m	soccer	partner (Bosu-ball)	1 picture	3 weeks<br>1.0 (1)<br>4.0 (4)<br>5.0 (5)	**3**	0	1	1	1	0	0	0	0
Macdonald et al. (2019) [36]	6	m	Gaelic football	partner	2 pictures	6 weeks<br>2.5 (1 - 3)	**3**	0	0	0	1	1	1	0	0

						2.8 (2 - 3)<br>8.1 (5 - 10)									
Marušič et al. (2020) [18]	18	m & f	recreationally active	custom-made device (elevated)	2 pictures	6 weeks<br>2.0 (2)<br>2.5 (2 - 3)<br>7.5 (5 - 8)	**6**	2	0	1	0	1	0	0	2
Matthews et al. (2017) [53] *strength protocol*	11	m	soccer	partner (thin mat)	1 picture	4 weeks<br>2.0 (2)<br>5.0 (5)<br>4.0 (4)	**3**	0	0	0	0	2	1	0	0
Matthews et al. (2017) [53] *endurance protocol*	9 (11)	m	soccer	partner (thin mat, rubber band assistance)	1 picture	4 weeks<br>2.0 (2)<br>5.0 (5)<br>12.0 (12)	**2**	0	0	0	0	1	1	0	0
Medeiros et al. (2020) [121] *low frequency*	15	m	soccer	partner (thin mat)	1 picture	8 weeks<br>1.0 (1)<br>3.4 (2 - 4)<br>8.7 (6 - 10)	**3**	0	0	0	0	1	0	0	2
Medeiros et al. (2020) [121] *high frequency*	17	m	soccer	partner (thin mat)	1 picture	8 weeks<br>2.0 (2)<br>3.4 (2 - 4)<br>8.7 (6 - 10)	**3**	0	0	0	0	1	0	0	2
Mendiguchia et al. (2015) [9]	27 (31)	m	soccer	not specified	N/A	7 weeks<br>0.6 (0 - 1)<br>2.3 (2 - 3)<br>5.6 (4 - 8)	**1**	0	0	0	0	1	0	0	0
Mendiguchia et al. (2020) [5]	7 (12)	m	soccer	partner	N/A	6 weeks<br>2.5 (1 - 3)	**1**	0	0	0	0	1	0	0	0

						2.8 (2 - 3)<br>8.1 - 9.0 (5 - 12)									
Mjølsnes et al. (2004) [12]	11	m	soccer	partner<br>(thin mat)	1 sketch<br>(2 positions)	10 weeks<br>2.7 (1 - 3)<br>2.9 (2 - 3)<br>9.0 - 9.4 (5 - 12)	**3**	0	0	0	0	1	0	0	2
Naclerio et al. (2013) [52]	10	m	soccer	partner	N/A	4 weeks<br>3.0 (3)<br>3.0 (3)<br>8.0 (8)	**2**	0	0	0	1	0	1	0	0
Navarro-Santana et al. (2020) [132]<br>*FIFA 11+*	18	m	soccer	partner	N/A	6 weeks<br>2.0 (2)<br>1.0 (1)<br>3.0 - 15.0 (3 - 15)	**0**	0	0	0	0	0	0	0	0
Nouni-Garcia et al. (2018) [15]	43 (46)	m	soccer	partner	N/A	2 seasons<br>N/A (2)<br>1.0 (1)<br>3.0 - 15.0 (3 - 15)	**0**	0	0	0	0	0	0	0	0
Olsen et al. (2005) [94]	958 (988)	m & f	handball	partner<br>(foam pad)	2 pictures	38 weeks<br>0.03 - 1.4 (1 - 5)<br>3.0 (3)<br>10.0 (10)	**1**	0	1	0	0	0	0	0	0
Owen et al. (2013) [92]	26	m	soccer	partner<br>(foam pad)	1 picture	29 weeks<br>2.0 (2)<br>2.5 (2 - 3)<br>6.8 (4 - 8)	**2**	0	1	0	0	1	0	0	0
Petersen et al. (2011) [13]	461	m	soccer	partner	1 sketch<br>(2 positions)	10 weeks<br>2.7 (1 - 3)	**3**	0	0	0	0	1	0	0	2

						2.9 (2 - 3)<br>9.0 - 9.4 (5 - 12)									
Pollard et al. (2019) [8]<br>*bodyweight*	10	m	recreationally active	NordBord prototype (elevated, foam pad)	3 pictures	6 weeks<br>1.3 (1 - 2)<br>3.0 (2 - 4)<br>5.3 (4 - 6)	**6**	2	1	1	1	1	0	0	0
Pollard et al. (2019) [8]<br>*weighted*	10	m	recreationally active	NordBord prototype (elevated, foam pad)	3 pictures	6 weeks<br>1.3 (1 - 2)<br>3.0 (2 - 4)<br>5.3 (4 - 6)	**7**	2	1	1	1	2	0	0	0
Presland et al. (2018) [7]<br>*low volume*	10	m	recreationally active	NordBord prototype (foam pad)	N/A	6 weeks<br>1.3 (1 - 2)<br>3.0 (2 - 4)<br>5.3 (4 - 6)	**8**	2	1	0	1	2	0	0	2
Presland et al. (2018) [7]<br>*high volume*	10	m	recreationally active	NordBord prototype (foam pad)	N/A	6 weeks<br>2.0 (2)<br>4.3 (4 - 5)<br>8.5 (6 - 10)	**7**	2	1	0	1	1	0	0	2
Rahlf et al. (2020) [133]<br>*pooled*	104 (342)	m	soccer	partner	N/A	42 weeks<br>2.0 (2)<br>1.0 (1)<br>3.0 - 15.0 (3 - 15)	**0**	0	0	0	0	0	0	0	0
Rey et al. (2017) [37]	16	m	soccer	partner	N/A	10 weeks<br>2.7 (1 - 3)<br>2.9 (2 - 3)<br>9.0 - 9.4 (5 - 12)	**3**	0	0	0	0	1	0	0	2
Ribeiro-Alvares et al. (2018) [47]	10	m & f	recreationally active	partner	N/A	4 weeks<br>2.0 (2)	**2**	0	0	0	1	1	0	0	0

						3.0 (3)<br>7.8 (6 - 10)									
Salci et al. (2013) [113]	13	f	recreationally active	partner	N/A	10 weeks<br>2.7 (1 - 3)<br>2.9 (2 - 3)<br>9.2 (5 - 12)	**1**	0	0	0	0	1	0	0	0
Sebelien et al. (2014) [60]	60 (72)	m	soccer	partner<br>(thin mat)	2 pictures	5 weeks<br>2.4 (1 - 3)<br>2.8 (2 - 3)<br>8.4 (5 - 12)	**1**	0	0	0	0	1	0	0	0
Seagrave et al. (2014) [80] *partner*	65 (243)	m	baseball	partner	N/A	25 weeks<br>sessions, sets and repetitions not specified	**0**	0	0	0	0	0	0	0	0
Seagrave et al. (2014) [80] *rigid fixation*	65 (243)	m	baseball	specialized equipment	3 pictures	25 weeks<br>sessions, sets and repetitions not specified	**2**	2	0	0	0	0	0	0	0
Severo-Silveira et al. (2018) [39] *constant training*	10 (19)	m	rugby	partner	1 picture	8 weeks<br>2.0 (2)<br>2.9 (2 - 3)<br>6.0 (6)	**3**	0	0	0	0	0	1	0	2
Severo-Silveira et al. (2018) [39] *progressive training*	11 (18)	m	rugby	partner	1 picture	8 weeks<br>2.0 (2)<br>3.4 (2 - 4)<br>8.4 - 9.0 (6 - 10)	**4**	0	0	0	0	1	1	0	2
Seymore et al. (2017) [57]	10	m & f	recreationally active	partner	N/A	6 weeks<br>2.5 (1 - 3)	**3**	0	0	0	0	1	0	0	2

						2.8 (2 - 3)<br>8.1 - 9.0 (5 - 12)									
Siddle et al. (2019) [24]	7 (8)	m	soccer, rugby	partner	N/A	6 weeks<br>1.8 (1 - 2)<br>2.7 (2 - 3)<br>7.3 (5 - 10)	**4**	0	0	0	1	1	0	0	2
Silvers-Granelli et al. (2015) [91]	675 (775)	m	soccer	partner	N/A	20 weeks<br>1.6 (N/A - 3)<br>1.0 (1)<br>3.0 - 15.0 (3 - 15)	**0**	0	0	0	0	0	0	0	0
Silvers-Granelli et al. (2017) [127]	675 (775)	m	soccer	partner	N/A	20 weeks<br>2.2 (2 - 3)<br>1.0 (1)<br>3.0 - 15.0 (3 - 15)	**0**	0	0	0	0	0	0	0	0
Slauterbeck et al. (2019) [119]	1825	m & f	football, soccer, basketball, lacrosse	partner	N/A	1 season<br>1.5 (N/A)<br>1.0 (1)<br>3.0 - 15.0 (3 - 15)	**0**	0	0	0	0	0	0	0	0
Small et al. (2009) [58]	16	m	soccer	partner	N/A	8 weeks<br>2.6 (1 - 3)<br>2.9 (2 - 3)<br>8.7 - 9.3 (5 - 12)	**3**	0	0	0	0	1	0	0	2
Soligard et al. (2008) [95]	1055	f	soccer	partner (foam pad)	1 picture	35 weeks<br>0.3 - 3.0 (N/A)<br>1.0 (1)<br>3.0 - 15.0 (3 - 15)	**1**	0	1	0	0	0	0	0	0
Steffen et al. (2008a) [93]	18	f	soccer	partner	N/A	10 weeks<br>0.0 - 2.9 (0 - 3)	**0**	0	0	0	0	0	0	0	0

						0.0 - 1.0 (0 - 1)<br>0.0 - 5.0 (0 - 5)									
Steffen et al. (2008b) [118]	1073 (1091)	f	soccer	partner	N/A	28 weeks<br>0.8 (N/A)<br>1.0 (1)<br>5.0 (5)	**0**	0	0	0	0	0	0	0	0
Suarez-Arrones et al. (2019) [26] *some experience*	16	m	soccer	not specified	N/A	17 weeks<br>1.4 (1 - 2)<br>2.9 (2 - 3)<br>9.1 (5 - 10)	**2**	0	0	0	1	1	0	0	0
Suarez-Arrones et al. (2019) [26] *large experience*	17	m	soccer	not specified	N/A	15 weeks<br>1.5 (1 - 2)<br>2.9 (2 - 3)<br>9.0 (5 - 10)	**2**	0	0	0	1	1	0	0	0
Tansel et al. (2008) [34]	15	m	basketball	partner	N/A	5 weeks<br>2.4 (1 - 3)<br>2.8 (2 - 3)<br>7.0 - 9.2 (5 - 12)	**1**	0	0	0	0	1	0	0	0
Trajkovic et al. (2020) [33]	19	m	soccer	partner	N/A	4 weeks<br>3.0 (3)<br>1.0 (1)<br>3.0 - 15.0 (3 - 15)	**0**	0	0	0	0	0	0	0	0
Tyler et al. (2017) [49]	50	m & f	not specified	partner	N/A	weeks not specified<br>3.0 (3)<br>3.0 (3)<br>8.0 - 12.0 (8 - 12)	**0**	0	0	0	0	0	0	0	0
Uysal et al. (2021) [140]	13 (14)	m	not specified	partner	N/A	8 weeks<br>2.6 (1 - 3)	**1**	0	0	0	0	1	0	0	0

						2.9 (2 - 3)<br>7.5 - 10.5 (5 - 12)									
van der Horst et al. (2015) [10]	292	m	soccer	partner	1 sketch<br>(2 positions)	13 weeks<br>1.9 (1 - 2)<br>2.9 (2 - 3)<br>8.3 (5 - 10)	**3**	0	0	0	0	1	0	0	2
Whyte et al. (2021) [65]	13	m	Gaelic football	partner<br>(thin mat)	3 pictures	4 weeks<br>2.0 (2)<br>3.3 (2 - 4)<br>7.8 (6 - 10)	**3**	0	0	0	1	1	0	1	0
Zein et al. (2020) [131]<br>*FIFA 11+*	13 (19)	m	soccer	partner	N/A	4 weeks<br>3.0 (3)<br>1.0 (1)<br>3.0 - 15.0 (3 - 15)	**0**	0	0	0	0	0	0	0	0

abbreviations: IQR, interquartile range; m, males; f, females; N/A, not available
